# What’s Good About Inclusion? An Ethical Analysis of the Ideal of Social Inclusion for People with Profound Intellectual and Multiple Disabilities

**DOI:** 10.1007/s10728-023-00470-y

**Published:** 2023-12-11

**Authors:** Simon van der Weele, Femmianne Bredewold

**Affiliations:** Department Citizenship and Humanisation of the Public Sector, University of Humanistic Studies, Utrecht, The Netherlands

**Keywords:** Social inclusion, Intellectual disability, Profound intellectual and/or multiple disabilities, Ethics

## Abstract

‘Social inclusion’ is the leading ideal in services and care for people with intellectual disabilities in most countries in the Global North. ‘Social inclusion’ can refer simply to full equal rights, but more often it is taken to mean something like ‘community participation’. This narrow version of social inclusion has become so ingrained that it virtually goes unchallenged. The presumption appears to be that there is a clear moral consensus that this narrow understanding of social inclusion is good. However, that moral consensus is not clear in the case of people with profound intellectual and/or multiple disabilities (PIMD), who are not able to express their needs and preferences verbally. Moreover, social inclusion has proven to be difficult to conceptualize and implement for people with PIMD. Therefore, it becomes imperative to ask about the ethical rationale of the narrow understanding of social inclusion. For what reasons do we think social inclusion is good? And do those reasons also apply for people with PIMD? This article addresses these questions by providing an ethical analysis of the ideal of social inclusion for people with PIMD. It discusses four ethical arguments for social inclusion and probes their relevance for people with PIMD. The article argues that none of these arguments fully convince of the value of the narrow understanding of social inclusion for people with PIMD. It ends with advocating for an ethical space for imagining a good life for people with PIMD otherwise.

## Introduction

‘Social inclusion’ is the leading ideal in services and care for people with intellectual disabilities in most countries in the Global North [45, 46, 66]. In these countries, the ideal of social inclusion informs disability policy and guides organizational and professional practice. Moreover, disability advocacy groups frequently utilize a vocabulary of social inclusion to communicate and frame their interests and demands. Social inclusion also figures widely in the UN United Nations Convention on the Rights of Persons with Disabilities [3]. In all, these societies imagine the good life for people with intellectual disabilities overwhelmingly in terms of social inclusion [45, 46, 66, 74].

It is possible to distinguish between two meanings of social inclusion. On the one hand, ‘social inclusion’ is a shorthand for the general principle of full equality of people with intellectual disabilities, including all associated rights. We call this the ‘broad’ understanding of social inclusion. On the other hand, ‘social inclusion’ can also refer to a specific understanding of what full equality for people with intellectual disabilities ought to look like in practice. In this second, narrower sense, social inclusion is typically imagined as something like this: people with intellectual disabilities are supported to live ‘in society’, to sustain relationships with people without intellectual disabilities, and to be present and participate in ‘the community.’ We call this the ‘narrow’ understanding of social inclusion.

In intellectual disability policy and scholarship, these two meanings of ‘social inclusion’ often intermingle, to the point where they have become seemingly synonymous. That is, full equality (the broad meaning) is almost uniformly believed to require community participation and interpersonal relationships (the narrow meaning). As a result, there are virtually no alternatives available to the narrow understanding of social inclusion for imagining the full equality of people with intellectual disabilities [66].

On the face of it, this lack of alternatives hardly seems a problem. The narrow understanding of social inclusion virtually goes unchallenged, in scholarly debates as in policy and practice [12, 15]. The presumption appears to be that there is a clear moral consensus amongst people with intellectual disabilities, service providers, policy makers, and scholars that social inclusion (in its narrow sense) is good. Indeed, even though various scholars have already argued or shown that this apparent moral consensus is in fact incomplete [1, 9, 12, 15, 21, 26, 33], the academic conversation on social inclusion is mostly about how to implement and measure this narrow understanding of social inclusion successfully—not about whether or why it is desirable.

However, the lack of alternatives to the narrow understanding of social inclusion becomes troubling when we consider a specific group of people with intellectual disabilities: namely, people with profound intellectual and multiple disabilities (PIMD). People with PIMD are individuals with ‘significant cognitive difficulties, with little or no apparent understanding of verbal language, little or no ability to care for oneself, and usually associated medical conditions’ [47, p. 265]. Very little is known about the social inclusion of this group to this day [25, 34, 47]. Some argue that they have been forgotten in discussions on inclusion [48].

The predicament faced by people with PIMD raises two problems for the narrow understanding of social inclusion. First, their complex needs make this narrow approach to social inclusion difficult to conceptualize and implement for this group [17, 73]: it is not immediately clear, for instance, what community participation might look like for someone with a profound intellectual disability who cannot talk or walk.

Second, the predicament faced by people with PIMD unsettles the moral consensus about social inclusion in its narrow sense. Grasping the needs and preferences of people with PIMD is exceedingly difficult and rife with ambiguities [27, 47, 49, 61, 63]. Put very simply, people with PIMD ‘cannot choose their own lifestyle’, as Clement and Bigby [17, p. 28] observe. As a result, for people with PIMD, the moral consensus is unclear because it is a group whose consensus cannot readily be obtained—certainly not in abstract terms. For these reasons, it is worrisome that no serious alternative interpretations of social inclusion seem available for people with PIMD.

The overlap between the two meanings of social inclusion makes the ideal of social inclusion (in its narrow sense) difficult to challenge: anyone who challenges the importance of community participation or interpersonal relationship seems to challenge the importance of full equality itself. However, when the narrow understanding of social inclusion seems unfit for people with PIMD, and when people with PIMD cannot unambiguously consent to it as the guiding ideal in shaping a good life for them, it becomes imperative to think about the ethical rationale of the narrow understanding of social inclusion. For what reasons do we think social inclusion is good? And do those reasons also apply for people with PIMD? In short, what is good about inclusion for them?

This question is deliberately provocative. By asking it, we want to break open the discussion about a good life for people with PIMD, which in our opinion is not asked often enough [80]. If ‘we’ (without intellectual disabilities) are primarily responsible for shaping a good life for people with PIMD, we must understand why it is ‘social inclusion’ that ought to guide our practices for doing so. To be clear, we are not questioning social inclusion in its broad sense, that is, as the full equality of people with PIMD. Rather, we are asking whether the ethical arguments for the narrowed *specification* of this ideal are sound when it comes to people with PIMD.

This article takes up this question by providing an ethical analysis of the ideal of social inclusion for people with PIMD. We discuss four ethical arguments for the narrow version of social inclusion and probe their relevance for people with PIMD. We take these arguments from the social-scientific literature on social inclusion. Although rarely developed in depth or even made explicit by the authors who provide them, they form the ethical backbone of the social inclusion literature. As we will argue, none of these arguments fully convince of the value or importance of social inclusion in its narrow sense for people with PIMD. In the conclusion, we therefore attempt to carve out an ethical space for imagining a good life for people with PIMD otherwise—a task that we cannot finish in this article, but that we invite other scholars to join us in.

First, we briefly outline the meanings of the term social inclusion and the ambiguities inherent in it. Second, we give a more detailed description of who we mean when we write of people with PIMD. Third, we present our ethical analysis. Finally, we begin the work of looking for alternatives to the narrow interpretation of social inclusion.

## The Ideal of Social Inclusion

As an ideal for shaping disability services, the term social inclusion has been in sway since at least the 1990s [20, 28]. Scholars typically regard social inclusion as a successor of the term ‘normalization’: the idea that people with intellectual disabilities ought to be supported to live ‘an existence as close to the normal as possible’ [53, p. 19]. The notion of normalization drove the deinstitutionalization movement, which from the 1950s onwards saw many people with intellectual disabilities move from large-scale and remote institutional facilities to small-scale living arrangements ‘in the community’ [26, 53, 62, 78]. According to Culham and Nind [20], social inclusion updates normalization by shifting emphasis from assimilation of *individuals* with disabilities to transformation of the *societies* in which they live to accommodate them. By now social inclusion has become the principal ideal through which societies in the Global North imagine a good life for people with intellectual disabilities.

As mentioned in the introduction, we can distinguish between social inclusion in a broad and narrow sense. In its broad sense, social inclusion refers to the idea of full equality of people with intellectual disabilities, including all associated rights. Taken in this way, social inclusion functions as a general ethical principle of sorts. In its narrow sense, social inclusion also refers to various specific ideas about the realization of equality in practice. Taken in this way, social inclusion invokes particular imaginations of the good life for people with intellectual disabilities.

Many researchers have formulated such narrower takes on social inclusion, often driven by the wish to render it fitter for implementation and measurement. Usually, such narrow understandings of social inclusion involve two elements: participation ‘in the community’ and the formation of personal relationships. For instance, based on a review of social inclusion literature, Simplican et al. [73, p. 18] have defined social inclusion as the ‘interaction between two major life domains: interpersonal relationships and community participation’. They point out that social-scientific studies on social inclusion typically utilize some version of this narrowed-down definition.

In practice, the broad and narrow senses of social inclusion have become nearly synonymous, as scholars rarely distinguish between them. As a result, social inclusion has become a notoriously diffuse and flexible term [2, 17, 18, 45, 56, 73, 81]: at once, it designates both an abstract ideal and a number of different, concrete policy ideas. This status quo leads to myriad ethical confusion. Scholars probing narrow specifications of social inclusion are mistakenly taken to question the broad ideal of equality for people with intellectual disabilities.

The result has been a deadlock in social inclusion research, as conceptual discussions about the term are stymied by fear of being labelled averse to full equality [12, 14, 15, 26]. Yet conceptual discussions about social inclusion are greatly needed when it comes to people with PIMD. To see why, we must first become clearer about who the individuals are who are labelled as such.

## People with Profound and/or Multiple Disabilities

The group of people with PIMD is notoriously difficult to define and there is no definition or classification enjoying universal agreement [40, 52, 80]. As some have pointed out, the act of labelling is in itself potentially antithetical to the ideal of inclusion—especially when labelling occurs in the language of lack and incapacity, as is often the case in descriptions of PIMD [35]. Nonetheless, for the sake of the clarity of our arguments, it is imperative to become more specific about the group under discussion.

Nakken and Vlaskamp [48, p. 85] speak of two ‘key defining characteristics’ of people with PIMD: first, ‘profound intellectual disabilities’, and second, ‘profound neuromotor dysfunctions’. This means that people with PIMD have little to no apparent understanding of verbal language and little to no ability to care for themselves [47, 48]. They also tend to have various medical conditions requiring regularly administered medication. Resultingly, people with PIMD have pervasive care needs, needing support for carrying out essentially every ordinary activity [51, 52]. In addition, due to their inability to communicate verbally, getting to know the needs and wants of people with PIMD tends to be exceedingly difficult [28, 40, 49, 61, 63].

Clearly, the specific needs and capacities of people with PIMD have implications for how they might realistically participate in society and develop relationships with other people [85]. Yet this simple fact is often treated with ambiguity by social inclusion researchers. Researchers appear hesitant to fully come to terms with the predicament [72] faced by people with PIMD, preventing them from appreciating the limits of the narrow understanding of social inclusion for this group. For instance, Talman et al. [, p. 85] write that ‘[i]t is troublesome that staff define what participation is for adults with PID or PIMD since research has shown that people with disabilities should be free to define participation for themselves’—as if people with PIMD could ever ‘define’ their preferred version of participation in any straightforward way. Similarly, Gauthier-Boudreault et al. note with some sense of understatement that ‘attending post-secondary education or being considered for paid employment 76*could* require substantial support from social, education, and community services’ for people with PIMD – as if a life without support is in any way imaginable for them [7, p. 249, emphasis ours]. We observe this tendency to downplay the predicament faced by people with PIMD in many other articles on social inclusion for people with PIMD [16, 17, 20, 68].

Of course, it is important not to ‘entrench low expectations’ of people with PIMD and acknowledge their capacity to learn and develop [38, 52, p. 2]. Moreover, we should not fall into the trap of thinking of people with PIMD exclusively in terms of lack and incapacity. Indeed, authors such as Kittay [38], Vorhaus [83], and Vehmas and Mietola [80] have provided beautiful thick descriptions of the ways people with PIMD engage joyfully with their environment and other people, and we have also witnessed this in our own extensive fieldwork in the care for people with PIMD [12, 88]. At the same time, we believe that taking seriously their predicament [72] demands we acknowledge their cognitive differences and the implications these may have for what living a good life means for them. It is for this reason we will now scrutinize the ethical discourse surrounding narrow understandings of social inclusion.

## Ethical Arguments for Social Inclusion

In this section, we analyze four ethical arguments for the narrow understanding of social inclusion most frequently found in the literature. For each argument, we provide a general characterization based on social inclusion literature. Then, we consider the argument’s merit and relevance in light of the lives of people with PIMD.

We must note that authors rarely invoke these arguments explicitly *as* ethical arguments for inclusion. More often, they are mentioned offhandedly, for instance to argue for the relevance of the research that is being reported. Hence, we are here rendering explicit ethical arguments that usually remain implicit. Moreover, while we have chosen to isolate these arguments here, they in fact often appear alongside one another in the articles we discuss. Our reason for isolating them, obviously, is to judge each by its own merits. Throughout, we mean the term ‘social inclusion’ to refer to its narrow understanding often found in the literature, unless we state otherwise.

### Social Inclusion is a Right

The first and most commonly found ethical argument for social inclusion is that social inclusion is a (human) right. Societies are therefore faced with an ethical mandate to accommodate people with intellectual disabilities and to assist them in achieving social inclusion [2, 6, 18, 20, 39, 52, 56, 57, 73, 76, 77, 89]. In this regard, authors frequently refer to the United Nations Convention on the Rights of Persons with Disabilities, which was drafted in 2006 and signed by 160 member states upon its opening in 2007 to explain the ethical necessity of social inclusion. For instance, Overmars-Marx et al. [56, p. 256] argue for the urgency of their inclusion research by insisting that the UN charter ‘reaffirms that all persons with all types of disabilities must enjoy all human rights and fundamental freedoms. One of the guiding principles is that people with disabilities have possibilities for full and effective participation and inclusion in society.’ Similarly, Amado et al. [2, p. 361] point at ‘the right to full participation and inclusion in society and community life’ inscribed in the UN Convention to explain why inclusion is important. Hence, by these authors and many others, the value of inclusion is explained by referring to inclusion as a (human) right.

Obviously, it is difficult to argue with the language of rights. That is why so many authors can invoke it without much further ethical explanation; invoking rights is enough to demonstrate the ethical urgency of inclusion. And there is no doubt, to our mind, that people with PIMD are rights-bearing subjects like all human beings.^1^ Yet in itself, the language of rights cannot fully account for the value of narrow understandings of social inclusion for people with PIMD.

First, *having* a right does not mean that one has to *use* that right. For instance, while the right to marriage is considered a human right, many choose not to use this right and live unmarried lives. In this sense, the right to marriage is of no import to them, even if they can rightly claim that they have it. It is therefore not self-evident that the existence of a right must mean that it will be of value to people with people with PIMD or that they will want to use it; especially since what they want is so difficult to ascertain.

Second, and more importantly, *having* a right says little about how that right ought to be *implemented*. This brings us back to the distinction between social inclusion in its broad and narrow sense, which most scholars fail to make. We might, with the UN Convention, affirm the broad meaning of social inclusion as full equality for people with PIMD, which the Convention phrases as ‘full and equal enjoyment of all human rights and fundamental freedoms by all persons with disabilities’ (Preamble). But we could still question whether the *specifications* of this ideal in the charter, for instance in terms of ‘full and effective participation and inclusion in society’ (Article 3) or ‘living independently and being included in the community’ (Article 19), works in practice for people with PIMD—whose pervasive support needs entail that they cannot ‘live independently’ nor participate ‘fully’ in society if we understand these terms in their ordinary meaning. In fact, the UN Convention leaves plenty of room for such questioning, a point we return to in the conclusion. The point here is that one can embrace the broad ideal of social inclusion for people with PIMD *and* also accept that it is unclear whether the narrow interpretation of this ideal is necessarily ‘right’ for people with PIMD.

The manner in which scholars employ rights-based argumentation reveals the ethical confusion at the heart of social inclusion research. Scholars use the *broad* right to social inclusion to ethically validate *narrow* interpretations of social inclusion, without being clear about this distinction or providing an ethical argument that explains why one should lead to the other. In effect, these two meanings of social inclusion become synonyms. This explains why discussions about social inclusion tend to get heated: anyone questioning narrow specifications of social inclusion seems to be questioning the general idea of full equality for people with intellectual disabilities itself. Yet these dynamics deter from serious discussion about the shape social inclusion ought to take for people with PIMD, for whom the narrow interpretation of social inclusion does not seem to work.

### Social Inclusion Improves Quality of Life

The notion that social inclusion improves the quality of life of people with intellectual disabilities is a second often-seen ethical argument for it [2, 4–6, 10, 22, 23, 25, 30, 39, 42, 43, 52, 73, 75, 81, 91]. Here, authors argue that social inclusion ‘confers some tangible benefit to the participant’ [21, p. 146]; typically, this benefit is phrased in terms of ‘quality of life’, but other terms such as ‘well-being’, ‘happiness’, and ‘health’ also occur. What is important is the idea that social inclusion makes the lives of people with intellectual disabilities better. McConkey [42, p. 207], for instance, writes that ‘[a]mong the consequences for residents in congregated settings [antithetical to social inclusion] has been a reduced quality of life… Moreover, the impact of social factors on a person’s health and well-being is increasingly recognized.’ Similarly, McCausland et al. [43, p. 880] indicate the relevance of their research by indicating that ‘[s]ocial inclusion is associated with improved well-being and quality of life for both the general and [intellectually disabled] populations.’ Again, this argument seems self-explanatory; after all, who would be against quality of life?

Certainly, a case can be made for correlating inclusion (as narrowly understood) with the quality of life or well-being of people with intellectual disabilities. Authors invoking the quality of life argument rightly cite evidence that social inclusion positively influences quality of life for people with intellectual disabilities [8, 23, 73, 93]. Indeed, social inclusion has long been one of the key domains in Schalock’s popular model for quality of life for people with intellectual disabilities [13, 71]. In this way, quality of life presents a strong ethical argument for the value of social inclusion for people with intellectual disabilities.

Yet again, things get more complex when it comes to the quality of life of people with PIMD. Although quality of life of people with intellectual disabilities has been widely researched, the quality of life of people with PIMD has received little attention in such research [40, 41, 60]. As a result, little is known about the correlation between quality of life of people with PIMD and social inclusion in its narrow sense. For instance, in a literature review on quality-enhancing interventions for people with profound intellectual and multiple disabilities, disabilities, Maes et al. [41, p. 174] did not find a single study on the quality of life domains of ‘community participation and rights’. Moreover, support workers and family members do not necessarily seem to prioritize these domains when asked about the quality of life of people with PIMD [51, 59]. In fact, some of them seem to worry that certain aspects of inclusion policies are harmful to the quality of life of their relative [12, 15]. Hence, the least we can say is that the correlation between quality of life of people with PIMD and social inclusion is as of yet not well-understood.

Further complicating things is that measuring quality of life of people with PIMD is challenging [58], particularly when it comes to subjective experience of quality of life [51]. Since people with PIMD cannot express themselves verbally, measuring their quality of life involves a reliance on proxies as well as a process of constant interpretation [55]. This raises a host of questions about the reliability of these measurements [50]. In particular, it makes it hard to ascertain how the domain of social inclusion affects subjective experiences of quality of life for people with PIMD.

To be sure, none of this disproves that promoting social inclusion in its narrow sense has a positive effect on the quality of life of people with PIMD. As such, the quality of life argument is not one we can reject outright. At the same time, we cannot unambiguously maintain it, either; it is less self-evident than it might at first appear to be.

### Social Inclusion is What People with Intellectual Disabilities Want

The third ethical argument for social inclusion is perhaps also the most straightforward: social inclusion is what people with intellectual disabilities want [2, 28, 43–45, 57]. Meininger [45, p. 192], for instance, writes of ‘the desire of many people with disability to be counted and to participate in their own living environment, in the social context of their immediate vicinity and in wider society’ to explain the ethical import of the ideal of inclusion. Similarly, McConkey [43, p. 207] indicates that ‘[p]eople with an intellectual disability (ID) often aspire to being more socially included. This finds expression in a desire to engage in more community activities… and to have more friends’. And McConkey et al. [44, p. 692] also describe social inclusion as ‘an aspiration often expressed by people with an [intellectual disability]’. Hence, these and other authors present the clear preference for social inclusion amongst people with intellectual disabilities as an ethical argument for pursuing and implementing it. If their desire to fully participate in society and develop relationship with non-disabled people is not enough of a reason to strive towards social inclusion, then what is?

Again, the desire for (some version of) social inclusion amongst people with intellectual disabilities is well-documented [31, 43, 67, 69]. As mentioned above, there does seem to be a rough moral consensus amongst people with intellectual disabilities, their relatives, support workers, policy makers and other relevant parties that social inclusion policies are rightly pursued. Yet as straightforward as this argument may sound when it comes to most people with intellectual disabilities, its validity for people with PIMD is far from obvious, for the same reason as the quality of life argument: it is simply not unambiguously clear nor easy to ascertain what people with PIMD want [47, 51, 80, 91]. Some argue we cannot even be sure whether people with PIMD can be said to have a ‘view’ in the first place—insofar as a ‘view’ requires some kind of intentional symbolic communication and conceptual understanding [86]. Evidently, the ‘moral consensus’ argument cannot be sustained for people with PIMD without this serious caveat.

As mentioned above, however, some social inclusion researchers seem to gloss over this point a little too quickly, writing as though such ambiguities should pose no serious problem for inclusion policies. Claims such as ‘it could be easy to determine what an adult with PID or PIMD wants if you know the person and understand his/her nonverbal communication’ [76, p. 83] problematically ignore the inevitable uncertainty involved with interactions involving people with PIMD [47]. Although the idea of moral consensus might superficially appear as the strongest ethical argument for social inclusion, then, it also most quickly loses its ethical force in the face of the predicament of PIMD.

### Social Inclusion Improves Society at Large

The final argument we want to discuss is that promoting social inclusion improves society as a whole. In this argument, policies of social inclusion are taken as a sign of moral progress, benefiting not only the lives of disabled people but society overall. Researchers have raised this point since the early days of deinstitutionalization: Nirje [53], in his founding text on normalization, opined that normalization would lead to a change in social attitudes towards people with intellectual disabilities that would not only improve their own lives for the better, but would also lead to improvements for their relatives, for support workers, and for society as a whole. This argument no longer receives as much explicit attention as the other three mentioned above, but it seems implicit in most conceptions of social inclusion, and other authors writing on inclusion do make similar comments [21]. For instance, Saxby et al. [70] write of benefits for what they call ‘abstract society’ following community participation of people with intellectual disabilities; and Amado et al. [2] suggest that community groups may become better when people with intellectual disabilities are present in them, as it can make these groups more caring and sensitive. The idea, in short, is that social inclusion is good for society.

Framing social inclusion as a matter of moral progress has philosophical credibility: for instance, moral philosophers like Axel Honneth [32] and Nancy Fraser [24] as well as many others have developed influential accounts of inclusion as moral progress. In the work of these authors, inclusive societies are better societies, for instance because they extend institutional recognition to a larger number of individuals (Honneth) or because they allow a larger number of individuals to participate ‘on par’ with one another (Fraser). To be sure, such philosophers are not defining social inclusion according to the narrow definition found in social inclusion literature. Nonetheless, there are clearly solid philosophical grounds for characterizing social inclusion as moral progress.

At the same time, the moral progress argument also has a flaw, which resides in its potential conflict with the argument about quality of life. Cummins and Lau have summarized the conflict as follows:

[The argument is that c]ommunity integration is good for future generations of people who are disabled. That is, community exposure changes public attitudes for the better, and this will enhance community acceptance as a long-term strategy. Even if it were so, and even if the evidence is weak and equivocal… ethical considerations demand that any imposed activity, such as community exposure, must be beneficial to the participants, not just to other people [21, p. 146].

In other words, Cummins and Lau believe the moral progress argument only holds insofar as it does not clash with the quality of life argument. This returns us to the difficulties encountered when appraising the latter argument: the challenge of determining the correlation between quality of life and social inclusion for people with PIMD and the concerns raised by proxies about the potential harms to their quality of life induced by social inclusion policies. Insofar as these difficulties remains unresolved, the weight of the argument of moral progress is up in the air. On its own, then, the moral progress argument seems to provide insufficient ethical grounding for taking the narrow understanding of social inclusion as model of the good life for people with PIMD.

## Conclusion: Re-imagining Social Inclusion

As we see it, none of the four ethical arguments for social inclusion we excavated from the literature are decisive about the ‘good’ of narrow definitions of social inclusion for people with PIMD. This conclusion largely comes down to two related points. First, it is extremely difficult to ascertain what people with PIMD ‘want’ from social inclusion and how it affects their subjective well-being or happiness. As we have seen, this point particularly troubles ethical arguments based on quality of life, moral consensus, or moral progress. Second, and consequently, it is unclear whether the narrow understanding of social inclusion is ‘good’ for people with PIMD. Granted that social inclusion is their right, there is still no clear ethical argument for assuming that it has to take the same shape as for people with milder intellectual disabilities. In this way, the ethical arguments usually provided for social inclusion as a model for the good life for people with intellectual disabilities come up short. It is worth asking, then, why so many social inclusion researchers stick with the narrow interpretation of social inclusion as a model for the good life for people with PIMD.

For an explanation, we must return to the failure of many scholars and policy makers to distinguish between social inclusion in its broad and narrow senses. As we already observed, the upshot of this failure is that anyone questioning the narrow specification of social inclusion risks being perceived as campaigning against the general idea of full equality for people with intellectual disabilities itself—and hence, as being labelled a bigot. In this ‘environment of moral judgment’ [14, p. 67], it is difficult and risky to open up ethical debate about the merits of the narrow understanding of social inclusion for groups such as people with PIMD. Yet as should be clear now, such debate is urgently needed, because this narrow understanding does not seem to fit their needs and capacities well.

To enable such debate, it is paramount to dislodge the general ideal of full equality for people with intellectual disabilities from its narrow specification in terms of participation and social relations. We can do so by turning to the work of political theorist Young [92]. Young famously argued that equality can be implemented either along the lines of the principle of sameness or the principle of difference. While the former regards equality as ‘treating everyone to the same standards, principles and rules’, the latter principle mandates ‘different treatment for disadvantaged and marginalised groups’ in order to respect their specific needs and capacities [92, p. 158]. Both principles can be followed to achieve equality; their application is not organized by rules, but depends on the social, political and cultural context.

Following Young, it is easy to see that the narrow interpretation of social inclusion relies on an interpretation of the principle of sameness. The logic is that equality is achieved when people with intellectual disabilities participate in society like non-disabled people and cultivate relations with non-disabled people. In other words, the idea is that equality for them consists in living lives that are the *same* as those of non-disabled people. This application of the principle of sameness is quite narrow, but it does fall within Young’s theory.

Scholars and policy makers alike have had good reason to draw on the principle of sameness to achieve social inclusion. The ideal of social inclusion was first developed to counter the dehumanizing and othering treatment experienced by many people with intellectual disabilities living in institutions. The idea was that people with intellectual disabilities are just as worthy of respect as all human beings, and thus to be treated *the same* as everyone else and to be enabled to live lives that are *the same* as everyone else’s. Hence, in the time when the ideal of social inclusion first emerged, it made sense to imagine it through the principle of sameness, and doing so has led to great improvements in the lives of people with intellectual disabilities.

However, as should now have become clear, the principle of sameness can only take us so far when it comes to achieving a good life for people with PIMD. Their needs and capacities do not seem served by imagining inclusion as participating in society ‘like everyone else’. Following Young, it is possible that implementing equality for people with PIMD warrants a *differentiated* treatment from other people with disabilities in order to do justice to their differences—which, in turn, could justify or even necessitate ideals that take this *difference* as a starting point [11, 12].

The principle of difference seems largely to have been forgotten in the social inclusion literature. Nonetheless, it is in fact also part of the UN Convention itself. For instance, the Convention claims to recognize ‘the diversity of people with disabilities’ and the ‘need to promote and protect the human rights of all persons with disabilities, including those who require more intensive support’ (Preamble, articles 9 and 10). However, the document speaks little of how ‘recognizing diversity’ might affect the implementation of a lofty ideal like inclusion.

One way in which some authors have already sought to do more justice to difference is by reimagining inclusion for people with PIMD (and also for people with intellectual disabilities more broadly) as *belonging* [18, 21, 29, 52, 64, 65]. Belonging makes space for difference by relinquishing the notion that a good life exclusively involves objective markers of inclusion, such as one’s amount of relationships with nondisabled people. Instead it emphasizes subjective feelings and experiences of being part of a larger community [29]. However, insofar as belonging designates a particular *experience*, the concept runs into many of the same problems as social inclusion when applied to the lives of people with PIMD. If belonging is to serve as a good life ideal for people with PIMD, researchers need to clarify what it might realistically mean for us to say that people with PIMD ‘feel’ like they ‘belong’. In this sense, belonging does not really solve any of the problems we highlighted, but only collects them under a different name.

Several moral philosophers have also attempted to formulate good life ideals for people with PIMD that start from difference: for instance, in terms of *dignity* [37, 82] and *flourishing* [54]. These ideals have barely been explored or even considered in the social-scientific discourse on intellectual disability, probably because they do not seem to follow the hallowed principle of sameness.

However, if we can dislodge the general ideal of full equality for people with intellectual disabilities from its narrow specification through the principle of sameness, an ethical space emerges for honestly debating how we might achieve a good life for people with PIMD otherwise. In such an ethical space, it is understood that social inclusion is not a prefabricated program, unburdened by uncomfortable ethical tensions and the ambiguities of context. Rather, it is understood that there are different routes to social inclusion; that social inclusion is an ethical puzzle that requires the careful balancing of the principles of sameness and difference, depending on the circumstances.

To achieve such an ethical space, we propose to alter the terms of the discussion: from ‘social inclusion’ to ‘a good life’. This is a shift we have already put to practice in this article. Insofar as research on social inclusion seeks to improve the lives of people with intellectual disabilities, it is always research on the good life, although researchers rarely make this explicit [87]. By speaking of the good life, we avoid confusing social inclusion as a broad ideal of equality with social inclusion as the narrow specification of this ideal. Moreover, asking about the good life sensitizes us to the decidedly *ethical* nature of discussions about inclusion, as it is in many ways the quintessential question of ethics [90].

We want to stress once more that we have no intention of suggesting that social inclusion is ‘bad’, or a ‘bad’ ideal, or that we are against equality, or that people with PIMD do not need relationships, or that presence in the community is bad for them. Our intention, rather, has been to carve open a space for reflecting seriously on a good life for people with PIMD, taking their specific needs and capacities as a point of departure. Young’s principle of difference is crucial here; and it is worth exploring its relevance not only for people with PIMD, but for people with intellectual disabilities more generally. As Stiker [74] pointed out so forcefully, how people with intellectual disabilities live their lives—and hence, whether they live good lives—largely hinges on the values cherished by the non-disabled majority. This is all the more true for people with PIMD, who have very few means for protesting against the way of life non-disabled people design for them. If equality is amongst the values we cherish, we must remain aware of this responsibility, and continue to probe our ethical reasons for pursuing one ideal and not another in on-going ethical reflection and discussion.

## Footnotes

1 The philosophical debate dealing with this question, centering on the ‘moral status’ of people with PIMD, lies beyond the scope of this paper. We will say that our sympathies lie with those in favour of moral status for people with PIMD [36, 79, 84]. For overviews of the debate, see Crary [19] and Vehmas and Curtis [79].
