# Correction: What’s Good About Inclusion? An Ethical Analysis of the Ideal of Social Inclusion for People with Profound Intellectual and Multiple Disabilities

**DOI:** 10.1007/s10728-024-00481-3

**Published:** 2024-02-02

**Authors:** Simon van der Weele, Femmianne Bredewold

**Affiliations:** Department Citizenship and Humanisation of the Public Sector, University of Humanistic Studies, Utrecht, The Netherlands

**Health Care Analysis**

10.1007/s10728-023-00470-y

In the sentence beginning of the second paragraph of the section ‘People with Profound and/or Multiple Disabilities’in this article, the text was inadvertently omitted should have read ’ Nakken and Vlaskamp [48,, p. 85] speak of two ‘key defining characteristics’ of people with PIMD: first, ‘profound intellectual disabilities’, and second, ‘profound neuromotor dysfunctions’. This means that people with PIMD have little to no apparent understanding of verbal language and little to no ability to care for themselves [47, 48]. They also tend to have various medical conditions requiring regularly administered medication. Resultingly, people with PIMD have pervasive care needs, needing support for carrying out essentially every ordinary activity [51, 52]. In addition, due to their inability to communicate verbally, getting to know the needs and wants of people with PIMD tends to be exceedingly difficult [28, 40, 49, 61, 63]’.

The original article has been corrected.

